# *GUCY2D* mutations in retinal guanylyl cyclase 1 provide biochemical reasons for dominant cone–rod dystrophy but not for stationary night blindness

**DOI:** 10.1074/jbc.RA120.015553

**Published:** 2021-01-13

**Authors:** Igor V. Peshenko, Elena V. Olshevskaya, Alexander M. Dizhoor

**Affiliations:** Pennsylvania College of Optometry, Salus University, Elkins Park, Pennsylvania, USA

**Keywords:** guanylate cyclase (guanylyl cyclase), retinal degeneration, cyclic GMP (cGMP), GUCY2D, photoreceptor, vision, calcium-binding proteins, RetGC, GCAP, RD3, signal transduction, stationary night blindness, cone–rod dystrophy, cyclic nucleotide, retina, neurodegenerative disease, congenital blindness

## Abstract

Mutations in the *GUCY2D* gene coding for the dimeric human retinal membrane guanylyl cyclase (RetGC) isozyme RetGC1 cause various forms of blindness, ranging from rod dysfunction to rod and cone degeneration. We tested how the mutations causing recessive congenital stationary night blindness (CSNB), recessive Leber's congenital amaurosis (LCA1), and dominant cone–rod dystrophy-6 (CORD6) affected RetGC1 activity and regulation by RetGC-activating proteins (GCAPs) and retinal degeneration-3 protein (RD3). CSNB mutations R666W, R761W, and L911F, as well as LCA1 mutations R768W and G982VfsX39, disabled RetGC1 activation by human GCAP1, -2, and -3. The R666W and R761W substitutions compromised binding of GCAP1 with RetGC1 in HEK293 cells. In contrast, G982VfsX39 and L911F RetGC1 retained the ability to bind GCAP1 *in cyto* but failed to effectively bind RD3. R768W RetGC1 did not bind either GCAP1 or RD3. The co-expression of *GUCY2D* allelic combinations linked to CSNB did not restore RetGC1 activity *in vitro*. The CORD6 mutation R838S in the RetGC1 dimerization domain strongly dominated the Ca^2+^ sensitivity of cyclase regulation by GCAP1 in RetGC1 heterodimer produced by co-expression of WT and the R838S subunits. It required higher Ca^2+^ concentrations to decelerate GCAP-activated RetGC1 heterodimer—6-fold higher than WT and 2-fold higher than the Ser^838^-harboring homodimer. The heterodimer was also more resistant than homodimers to inhibition by RD3. The observed biochemical changes can explain the dominant CORD6 blindness and recessive LCA1 blindness, both of which affect rods and cones, but they cannot explain the selective loss of rod function in recessive CSNB.

Two isozymes of retinal membrane guanylyl cyclase, RetGC1 and RetGC2 (1, 2, 3, 4), produce cGMP in the outer segments of vertebrate photoreceptors via negative Ca^2+^ feedback. The inward current carried by Na^+^ and Ca^2+^ influx through cGMP-gated channels partially depolarizes rods and cones in the dark (Refs. 5, 6, 7; reviewed in Refs. 8, 9, 10). Light activates cGMP hydrolysis by phosphodieterase-6 and hyperpolarizes photoreceptors by closing cGMP-gated channels (reviewed in Refs. 8, 9, 10, 11, 12). Ca^2+^/Mg^2+^-binding proteins, GCAPs (13, 14, 15, 16), respond to the interruption of Ca^2+^ influx through the channels by converting into a Mg^2+^-liganded state (16) and accelerate cGMP synthesis by RetGC, thus expediting the recovery of rods and cones from excitation and allowing them to adapt to light (17, 18, 19). Once the cGMP-gated channels reopen in the dark and the influx of Ca^2+^ is restored, Ca^2+^ GCAPs decelerate RetGC activity (reviewed in Refs. 12 and 16). Unlike GCAPs, RD3 protein (20, 21) is a Ca^2+^-insensitive inhibitor of RetGC (22, 23), and it does not affect the Ca^2+^ feedback regulation of cyclase (22). Instead, RD3 helps rods and cones to accumulate RetGC in the outer segment (21, 24) and also protects them from degeneration (20, 25), possibly by suppressing RetGC activation in the inner segment of photoreceptor (24, 25, 26, 27).

Numerous mutations in GCAP1, RD3, and RetGC1 have been linked to congenital blindness (reviewed in Refs. 9, 21, and 28, 29, 30, 31, 32). Mutations in *GUCY2D* gene coding for a human RetGC1 cause various forms of blindness, ranging from selectively disabling rod responses to dim light to complete blindness via degeneration of rods and cones. Substitutions of Arg^838^ in the RetGC1 dimerization domain cause autosomal dominant cone–rod dystrophy type 6 (CORD6), a rapidly progressing loss of vision caused by the degeneration of functional cones and rods (33, 34, 35). The CORD6-linked mutations reduce the sensitivity of the GCAP/RetGC1 complex to deceleration by Ca^2+^ (34, 35, 36). Consequently, the excessive production of cGMP in the dark elevates the influx of Na^+^ and Ca^2+^ in photoreceptors and provokes their apoptosis (37, 38).

Various nonsense and missense *GUCY2D* mutations in RetGC1 cause recessive blindness, Leber's congenital amaurosis type 1 (LCA1) (30, 31), where most rods and cones remain alive but are dysfunctional from birth (39). The LCA1-linked mutations in the intracellular portion of the cyclase eliminate or strongly reduce the activity of RetGC1 *in vitro* (39). Recessive *GUCY2D* mutations were also recently reported to cause congenital stationary night blindness (CSNB) (40). Unlike LCA1, CSNB patients retain daylight (cone-specific) vision but lack dim light (rod-specific) vision. Stunkel *et al.* (40) identified four CSNB *GUCY2D* alleles, three of which coded for mutations in a cytoplasmic portion of RetGC1. Notably, in every reported case the recessive CSNB allele was accompanied by a recessive *GUCY2D* LCA1 allele.

In the present study, we analyzed how the activity and regulatory properties of RetGC1 were affected by substitutions in the cytoplasmic portion of the enzyme, causing different types of blindness (Fig. 1). As expected, two LCA1 RetGC1 mutants completely lacked GCAP-stimulated activity. Surprisingly, however, the three tested CSNB-linked mutants were also inactive when expressed individually or co-expressed with the LCA1 variants accompanying them in CSNB patients. We also found that the CORD6 substitution, R838S, dominates Ca^2+^ sensitivity in the cyclase heterodimer containing a WT subunit. This can further explain the strong dominant phenotype of the mutations, leading to the abnormal Ca^2+^ sensitivity of the cGMP production that is typical for this disease (33, 34, 35, 36).

**Figure 1:**
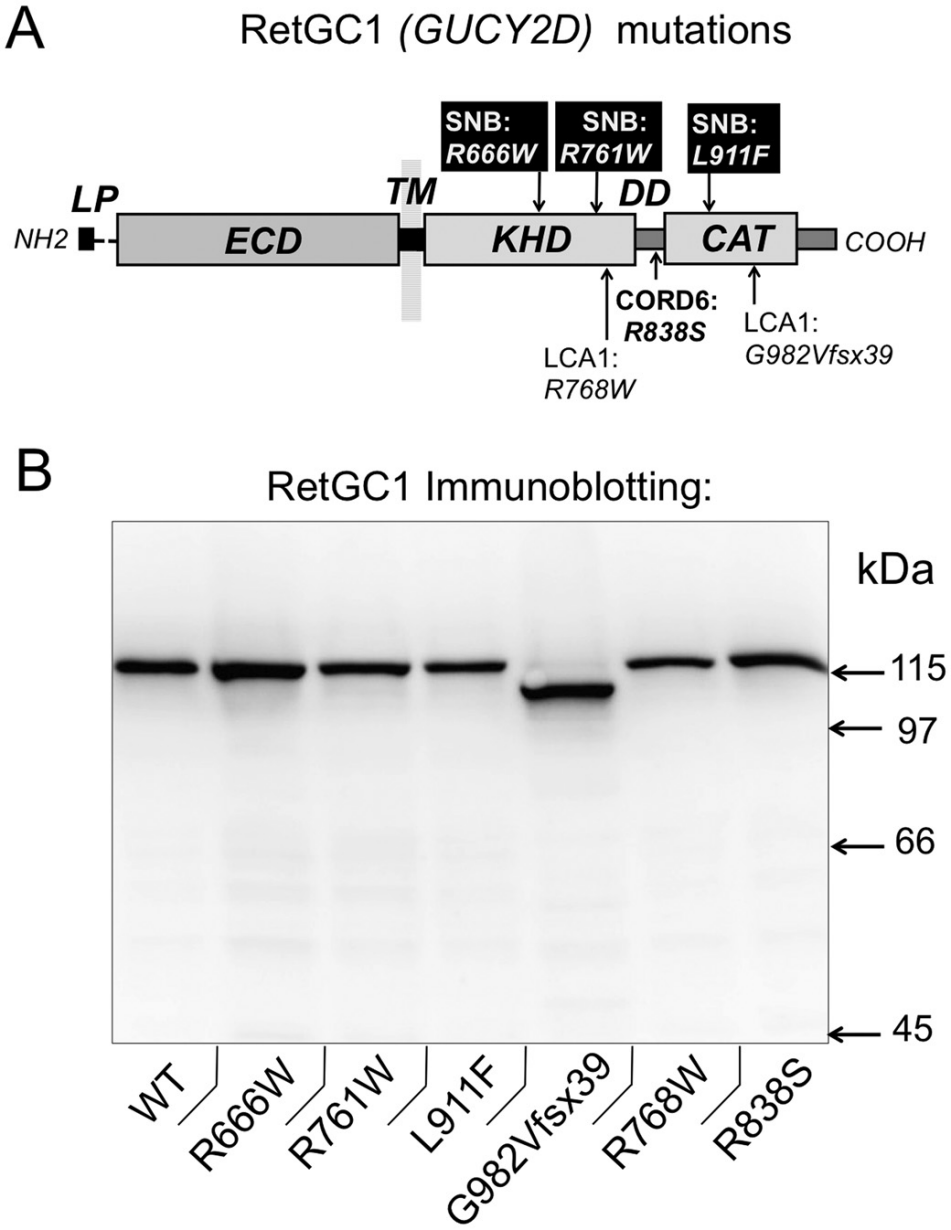
**Human RetGC1 (GUCY2D) mutations analyzed in this study.***A*, the diagram of RetGC1 primary structure (1, 2, 4). *LP*, leader peptide; *ECD*, extracellular domain; *TM*, transmembrane segment; *KHD*, kinase homology domain; *DD*, dimerization domain; *CAT*, catalytic domain. *Arrows* indicate the positions of mutations causing blindness: the R768W substitution and G982VfsX39 frameshift/truncation cause LCA1 (30, 39, 41); the R666W, R761W, and L911F substitutions cause CSNB (40); and the R838S substitution causes dominant cone–rod dystrophy CORD6 (34). *B*, Western immunoblotting of RetGC1 variants expressed in HEK293 cells as described under “Experimental procedures.”

## Results

### RetGC1 variants harboring GUCY2D CSNB, LCA1, and CORD6 mutations

The primary structure of RetGC1 includes several domains homologous to other membrane guanylyl cyclases (Fig. 1*A*). The “extracellular” domain, located in the intradiskal space of the photoreceptor disks, connects with the cytoplasmic portion of the cyclase via a short transmembrane region. The cytoplasmic part of the enzyme includes a protein kinase homology domain, a catalytic domain, and a short dimerization domain located between the kinase homology and the catalytic domains (2, 4, 32). The *GUCY2D* CSNB alleles encode substitutions: R666W or R761W, in the RetGC1 kinase homology domain or L911F in the catalytic domain (40). The CSNB alleles R761W and L911F were both accompanied by the LCA1 allele R768W, and the R666W allele was accompanied by the LCA1 allele G982VfsX39 (also known as c.2943delG or p.S981del1bp) (30, 39, 40, 41). Unlike the recessive LCA1 and CSNB mutations, the CORD6-linked R838S substitution in RetGC1 dimerization domain causes degeneration of heterozygous photoreceptors (33, 34, 35). All these RetGC1 variants in our study were expressed in HEK293 cells, and immunoblotting of the membrane fractions isolated from the transfected cells confirmed the presence of RetGC1 polypeptides of ∼105 kDa in the case of G982VfsX39 and of 115 kDa for all other variants (Fig. 1*B*).

### GCAP1 and RD3 binding to RetGC1 mutants in cyto

**Figure 2:**
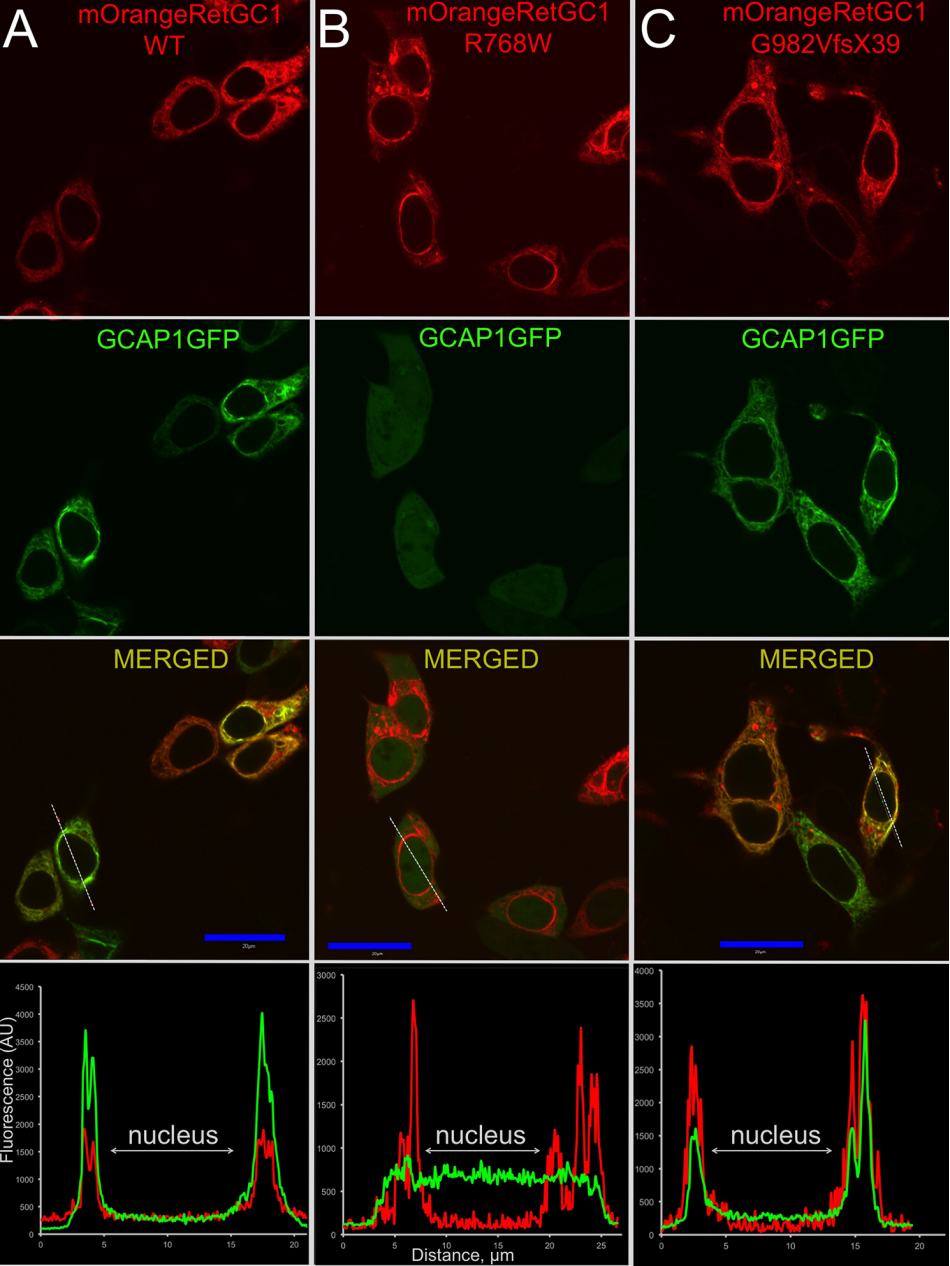
**Localization of LCA1 RetGC1 and GCAP1 co-expressed in HEK293 cells.***A–C*, representative confocal images of GCAP1-GFP (*green*) and the mOrange-tagged WT (*A*), R768W (*B*), and G982VfsX39 (*C*) RetGC1 (*red*). *Blue bar*, 20 μm. The distribution of the two fluorochromes across the cells along the *dashed line* in the respective merged images are shown in the *bottom panels*. Note the co-localization patterns in the cases of WT and G982VfsX39 RetGC1 and the lack of co-localization in R768W RetGC1. The PCC values are summarized in Table 1. The other details are described under “Experimental procedures.”

**Figure 3:**
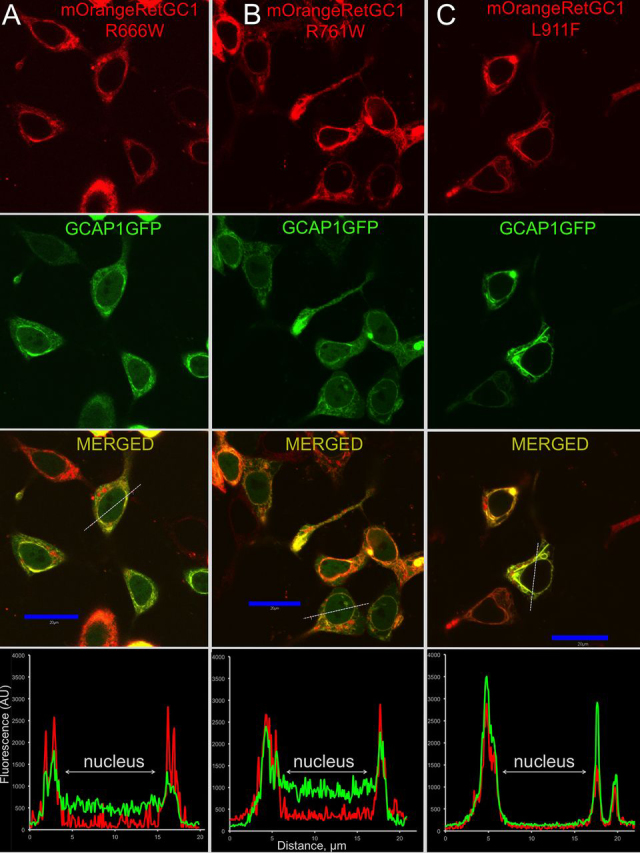
**Localization of CSNB RetGC1 and GCAP1 co-expressed in HEK293 cells.** The mOrange-tagged CSNB RetGC1 mutants R666W (*A*), R761W (*B*), and L911F (*C*) were co-expressed with GCAP1-GFP and analyzed as described in Fig. 2 and under “Experimental procedures.” Note the clearer co-localization pattern in the case of L911F and the less clearly defined patterns in the cases of R666W and R761W RetGC1. The PCC values are summarized in Table 1.

**Figure 4:**
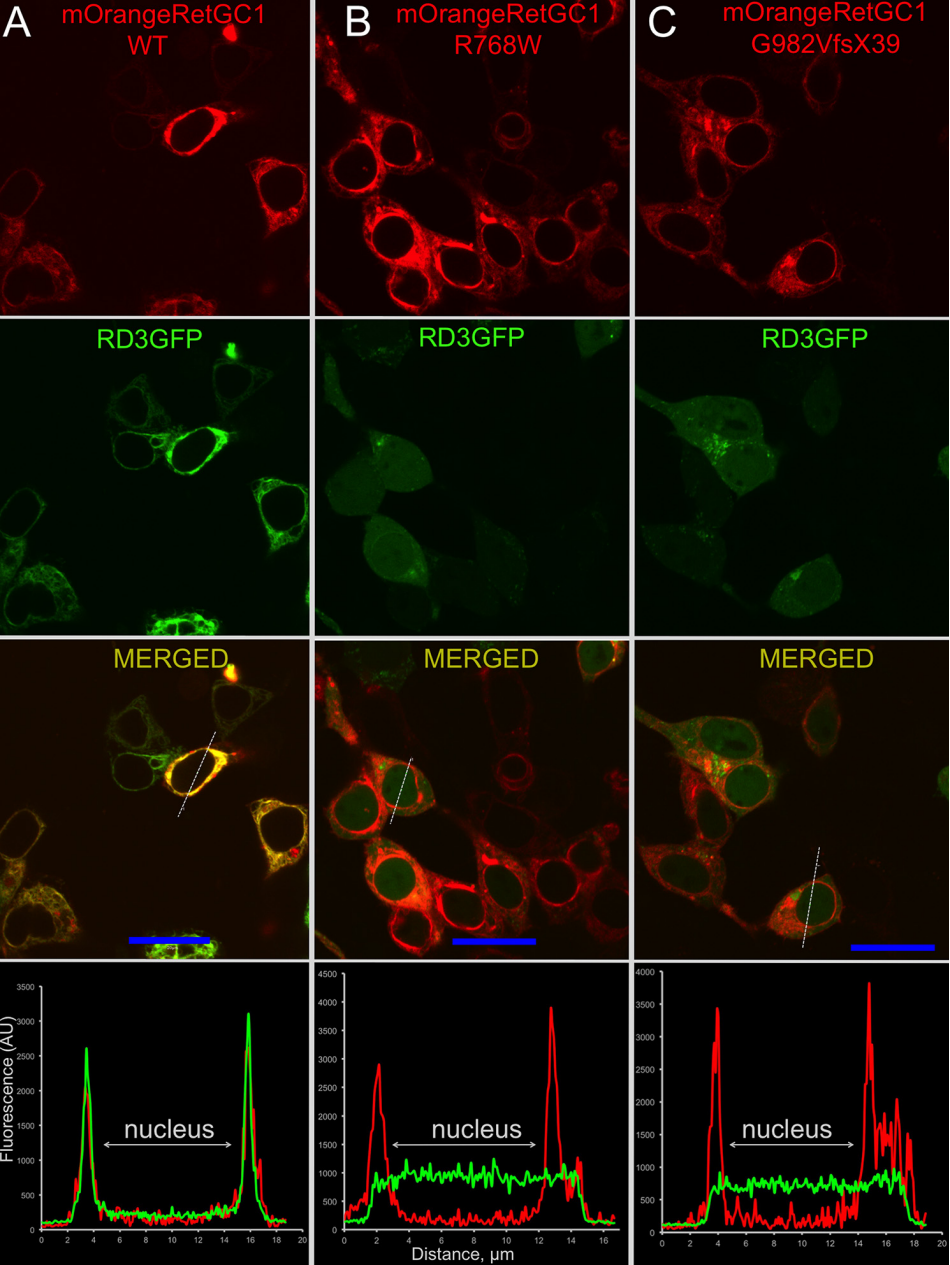
**Localization of RD3 and LCA1 RetGC1 co-expressed in HEK293 cells.***A–C*, representative confocal images of RD3-GFP (*green*) and mOrange-tagged (*red*) WT RetGC1 (*A*), R768W (*B*), and G982VfsX39 (*C*). The cells were transfected and analyzed as described under “Experimental procedures.” Note the well-defined RD3 co-localization with the cyclase in case of WT RetGC1 and the lack of co-localization in the cases of both LCA1 mutants. The PCC values are summarized in Table 2.

**Figure 5:**
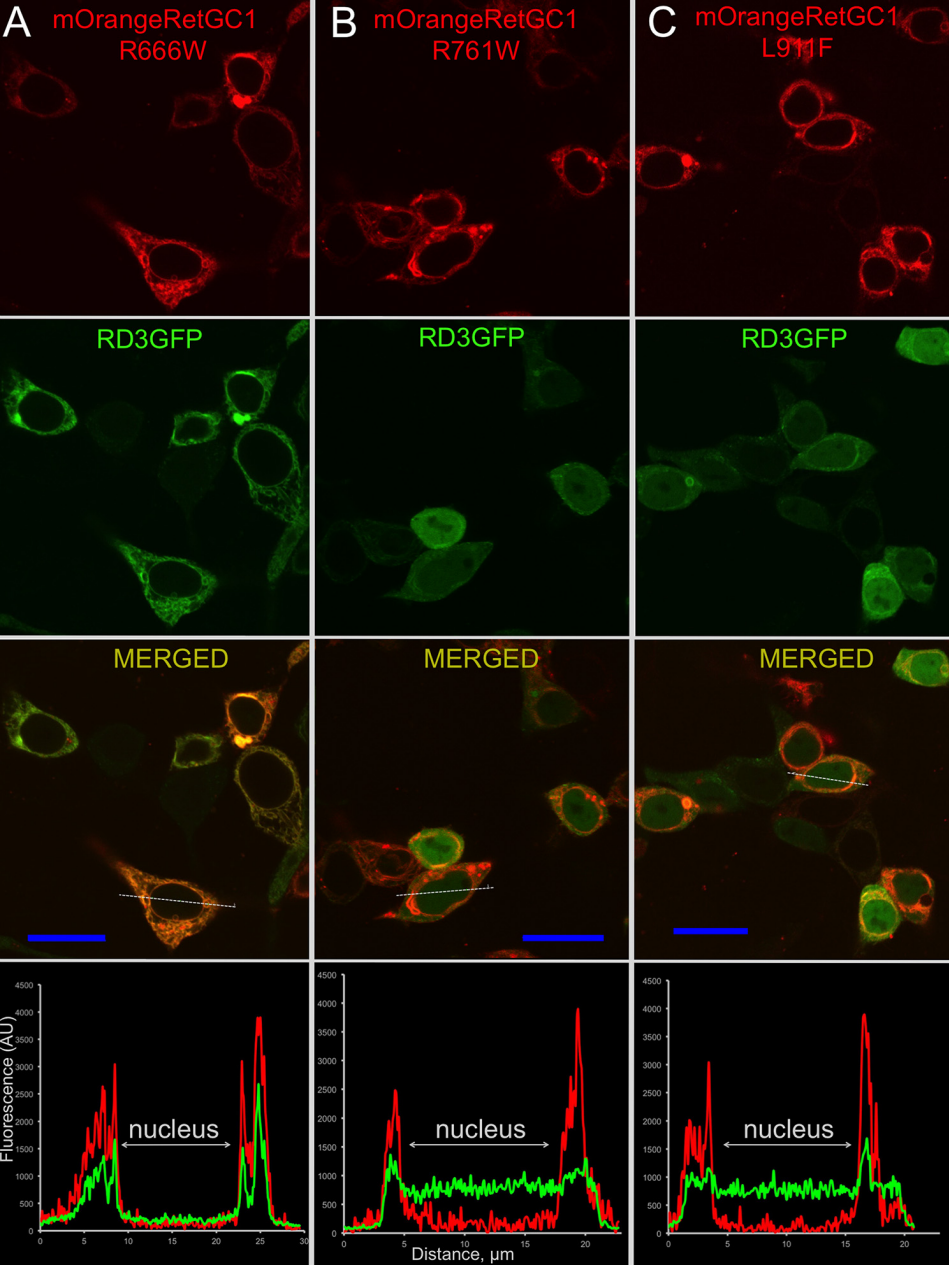
**Localization of RD3 and CSNB RetGC1 co-expressed in HEK293 cells.***A–C*, the representative confocal images of mOrange-tagged R666W (*A*), R761W (*B*), and L911F (*C*) RetGC1 co-expressed with RD3-GFP. Note the well-defined RD3 co-localization with R666W RetGC1 and its poor co-localization with the R761W or L911F RetGC1. The PCC values are summarized in Table 2.

We tested how the LCA1 and CSNB mutations affected RetGC1 association with its regulatory proteins. Biochemical testing of such protein complexes by conventional pulldown or co-immunoprecipitation techniques could not be applied to studying the RetGC–GCAP complex, because it disintegrates in detergents (42). Therefore, we employed a previously characterized method of co-localization in living HEK293 cells (43, 44, 45) using co-expression of mOrange-tagged RetGC1 with its GFP-tagged regulatory proteins (Figure 2, Figure 3, Figure 4, Figure 5). GCAP1-GFP and RD3-GFP expressed in the absence of RetGC1 are diffusely spread throughout the cytoplasm and the karyoplasm (43, 44, 45), but when co-expressed with the mOrange-RetGC1, they co-localize with the membrane cyclase, mostly in the endoplasmic reticulum (43, 44, 45). In contrast to WT (Fig. 2*A*), the LCA1 R768W RetGC1 (Fig. 2*B*) failed to bind GCAP1 *in cyto* (Table 1). However, GCAP1 co-localized with the G982VfsX39 LCA1 RetGC1 in a manner very similar to the WT RetGC1 (Fig. 2*C* and Table 1). CSNB mutations also differentially affected the ability of RetGC1 to bind with GCAP1 *in cyto* (Fig. 3). The co-localization of GCAP1 with R666W and R761W RetGC1 was visibly compromised (Fig. 3, *A* and *B*, and Table 1), whereas its co-localization with the L911F RetGC1 was not significantly different from the WT RetGC1 (Fig. 3*C* and Table 1). The ability to associate *in cyto* with RD3 also varied between different LCA1 and CSNB mutants. It was clearly presented in the R666W RetGC1 but became completely undetectable in R768W or strongly compromised in other mutants, such as R761W, L911F, and G982VfsX39 (Figure 4, Figure 5 and Table 2). The CORD6 substitution, R838S, did not compromise the *in cyto* RetGC1 binding with GCAP1 or RD3 (Table 1, Table 2).

**Table 1:** PCC for co-localization of RetGC1 with GCAP1 variants *in cyto* The mOrange-RetGC1 was co-expressed in HEK293 cells with GCAP1-GFP as described under “Experimental procedures.” The PCC was determined using confocal microscopy and Olympus FluoView FV10-ASW software (45, 59). Statistically significant differences from wildtype (ANOVA/Bonferroni post hoc test, confidence level 99%) are highlighted in *bold*. Note that PCC = 1.0 for the theoretical complete co-localization of two fluorochromes, whereas PCC < 0.6 indicates lack of co-localization (67).

Mutation	PCC (means ± S.D., *n*)	*P* (ANOVA/Bonferroni)
**WT**	0.900 ± 0.054, 23	
**CSNB mutations**		
R666W	**0.64 ± 0.15,** 37	**<0.0001**
R761W	**0.64 ± 0.14,** 33	**<0.0001**
L911F	0.89 ± 0.06, 33	1
**LCA1 mutations**		
R768W	**0.11 ± 0.10,** 27	**<0.0001**
G982VfsX39	0.87 ± 0.05, 26	1
**CORD6 mutation**		
R838S	0.89 ± 0.05, 25	1

**Table 2:** Co-localization of RetGC1 variants with RD3 *in cyto* The PCC values for co-localization of the mOrange-RetGC1 and RD3-GFP co-expressed in HEK293 cells (45, 59, 60). Statistically significant (ANOVA/Bonferroni post hoc test) differences from wildtype are highlighted in *bold.*

Mutation	PCC, Mean ± S.D., *n*	*P* (ANOVA/Bonferroni)
**WT**	0.91 ± 0.03, 29	
**CSNB mutations**		
R666W	0.90 ± 0.03, 26	1
R761W	**0.50 ± 0.15,** 31	**<0.0001**
L911F	**0.60 ± 0.10**, 37	**<0.0001**
**LCA1 mutations**		
R768W	**0.27 ± 0.17,** 32	**<0.0001**
G982VfsX39	**0.43 ± 0.09**, 32	**<0.0001**
**CORD6 mutation**		
R838S	0.91 ± 0.04, 27	1

### CSNB mutations suppress RetGC1 activation by human GCAPs

**Figure 6:**
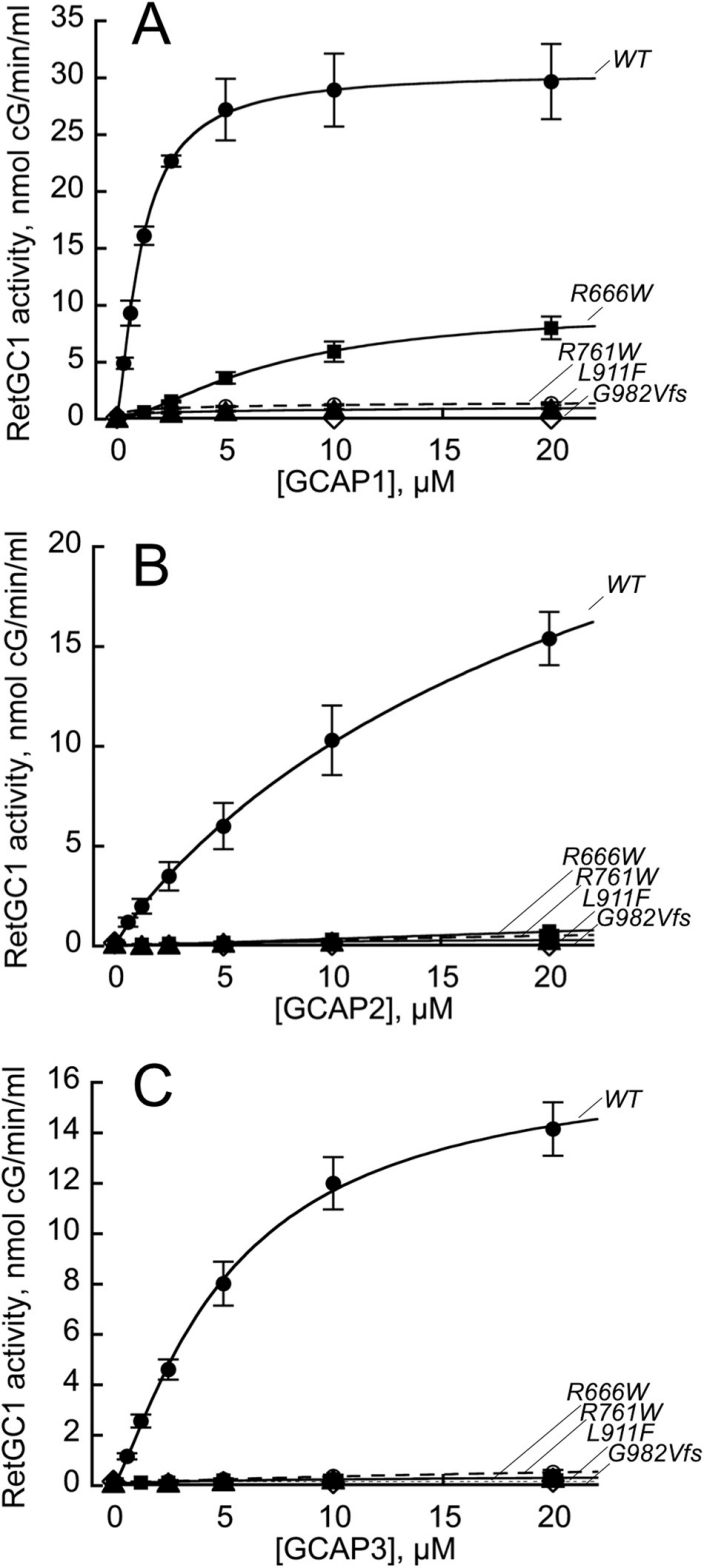
**LCA1 and CSNB RetGC1 mutations disable activation of RetGC1 by Mg^2+^GCAPs.***A–C*, the guanylyl cyclase activity (means ± S.D., three independent measurements) in the HEK293 membranes reconstituted with purified recombinant human GCAP1 (*A*), GCAP2 (*B*), or GCAP3 (*C*) in the presence of 10 mm Mg^2+^ and 2 mm EGTA: *filled circles*, WT RetGC1; *filled square*, R666W; *open circle*, R761W; *filled triangle*, L911F; *open diamond*, G982VfsX39. The data were fitted assuming a sigmoidal function, *A* = *A*_0_/(1 + ([GCAP]_1/2_/[GCAP])*^h^*), where *A*_0_ is the maximal cyclase activity, [GCAP] is the concentration of GCAPs in the assay, and *h* is the Hill coefficient. See “Experimental procedures” for other details.

Mg^2+^ GCAP1, a ubiquitous activator of RetGC1 in rods and cones, stimulated WT human RetGC1 *in vitro* ([GCAP]_1/2_ = 1.13 ± 0.04 μm) (Fig. 6*A*), but not the LCA1 RetGC1 variants (30, 39, 41, 46) coded by the second *GUCY2D* alleles in CSNB patients (40), R768W and G982VfsX39 (Fig. 6). GCAP2, the ancillary activator of RetGC in rods (14, 47, 48), and GCAP3, an isoform expressed exclusively in a subset of cones (49, 50), both stimulated WT human RetGC1, albeit with a lower apparent affinity than GCAP1 ([GCAP]_1/2_ = 19 ± 2.4 μm, and [GCAP]_1/2_ = 5.3 ± 0.9 μm, respectively). However, they also completely failed to activate the two LCA1 RetGC1 variants (Fig. 6, *B* and *C*). It is worth pointing out here that the inability of a human Mg^2+^ GCAP2 to activate WT human RetGC1 *in vitro* reported in Ref. 56 was most likely due to a deficiency in the preparation of a functional GCAP2.

**Figure 7:**
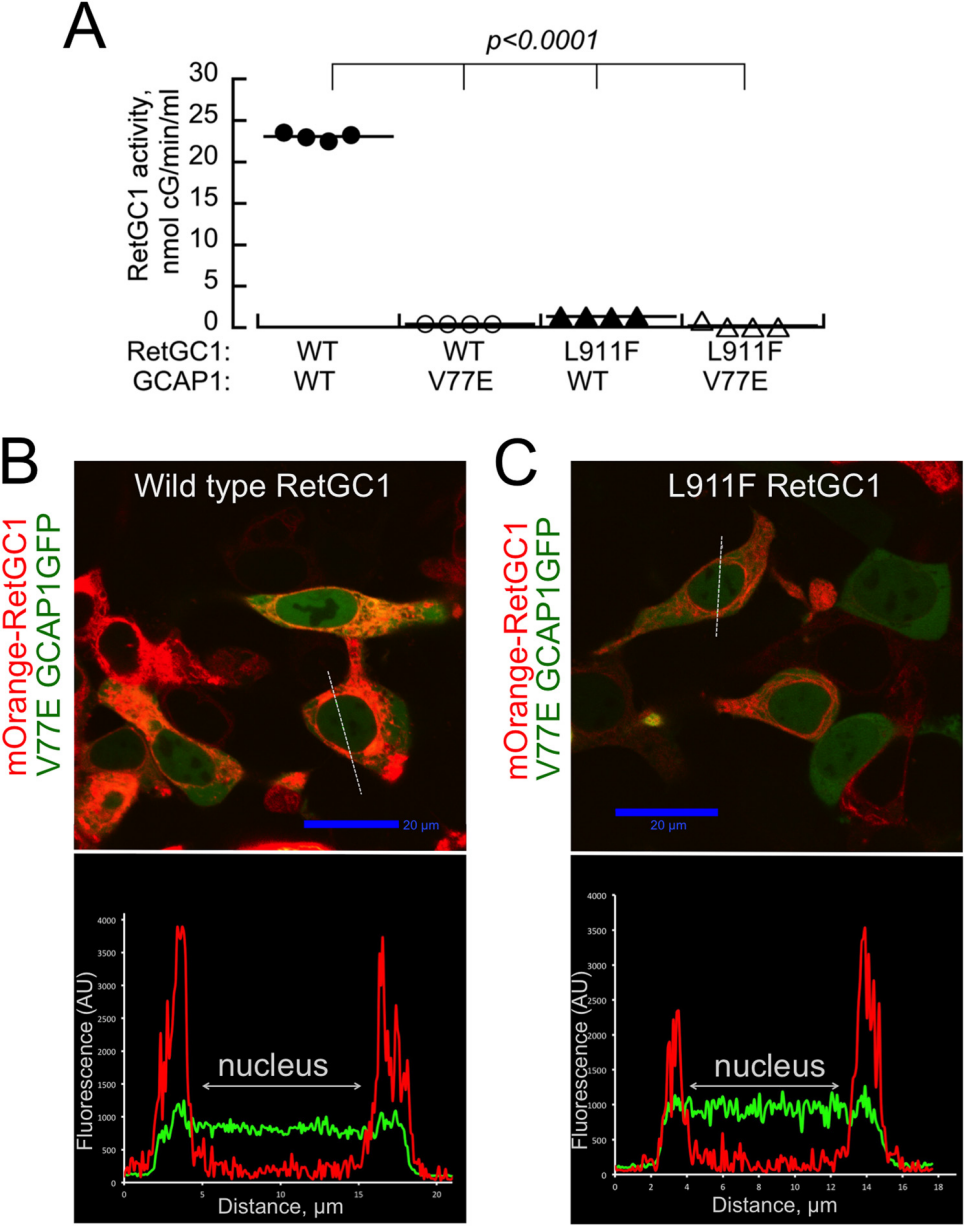
**L911F RetGC1 does not bind V77E GCAP1.***A*, guanylyl cyclase activity in HEK293 membranes expressing WT (*circles*) or L911F (*triangles*) RetGC1 reconstituted with the WT (*filled symbols*) or the V77E (*open symbols*) GCAP1 (43). *B* and *C*, representative merged confocal images of the mOrange-tagged WT (*B*) and L911F (*C*) RetGC1 co-expressed with V77E GCAP1-GFP. The respective *bottom panels* show the distribution of the two fluorochromes across the cells along the *dashed lines*. The respective PCC values (0.43 ± 0.19, *n* = 29, and 0.33 ± 0.12, *n* = 27) indicated the lack of co-localization. Compare with Figs. 2*A* and 3*C* and Table 1.

Surprisingly, none of the mutants coded by CSNB-specific alleles was efficiently activated by GCAPs (Fig. 6, *A–C*). A low level of activity was detectable in GCAP1-stimulated R666W RetGC1, but the apparent affinity for GCAP1 was strongly reduced ([GCAP1]_1/2_ = 7.25 ± 0.83 μm *versus* 1.13 ± 0.04 μm in WT; *p* = 0.006, Student's *t* test). The R761W and L911F RetGC1 were completely inactive in the presence of all three GCAP isoforms. The lack of activity in the L911F RetGC1 (Fig. 6*A*) appeared to be at odds with the clearly defined GCAP1 co-localization pattern *in cyto* (Fig. 3*C*). To ensure that the GCAP1-GFP binds with the L911F RetGC1 *in cyto* specifically via GCAP1 rather than GFP moiety, we used V77E GCAP1 lacking the ability to bind WT RetGC1 (43) as a control. The L911F RetGC1 did not co-localize with the V77E GCAP1 *in cyto* (PCC = 0.33 ± 0.12, *n* = 27) (Fig. 7).

### CSNB and LCA1 mutations in RetGC1 do not complement each other

**Figure 8:**
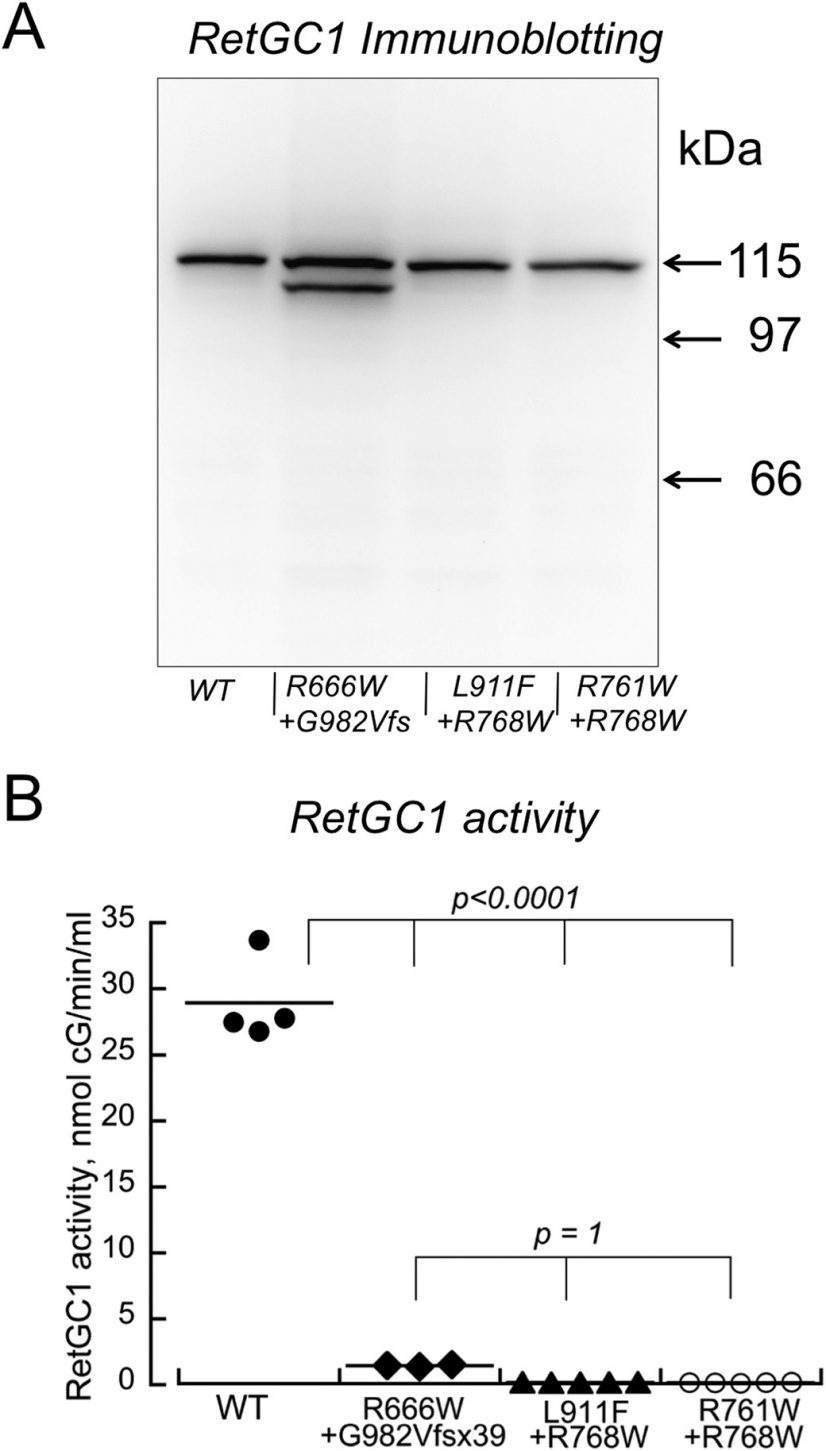
**CSNB *GUCY2D* allelic combinations fail to restore RetGC1 activity.***A*, Western immunoblotting of CSNB and LCA1 RetGC1 co-expressed in HEK293 cells matching the *GUCY2D* allelic combinations in CSNB patients (40); the lower band in the R666W1G982fsX39 sample belongs to G982VfsX39 RetGC1 (see Fig. 1). *B*, RetGC1 activity in the presence of 10 mm GCAP1, 10 mm Mg^2+^, and 2 mm EGTA; WT (*filled circles*), R666W1 G982fsX39 (*filled diamonds*), L911F1 R768W (*filled triangles*), and R761W 1 R768W (*open circles*). The p values are from ANOVA/Bonferroni post hoc test).

The inactive CSNB RetGC1 mutants could hypothetically create functional heterodimers in CSNB patients using the respective *GUCY2D* LCA1 allele products (40). Therefore, we co-expressed in HEK293 cells the pairs of CSNB and the LCA1 RetGC1 variants, mimicking their allelic combinations in CSNB patients: R666W + G982VfsX39, R761W + R768W, and L911F + R768W (Fig. 8). No cyclase activity was detected in any of the three combinations, arguing against functional complementation between the LCA1 and CSNB alleles.

### Ca^2+^ sensitivity in heterodimers containing R838S RetGC1

**Figure 9:**
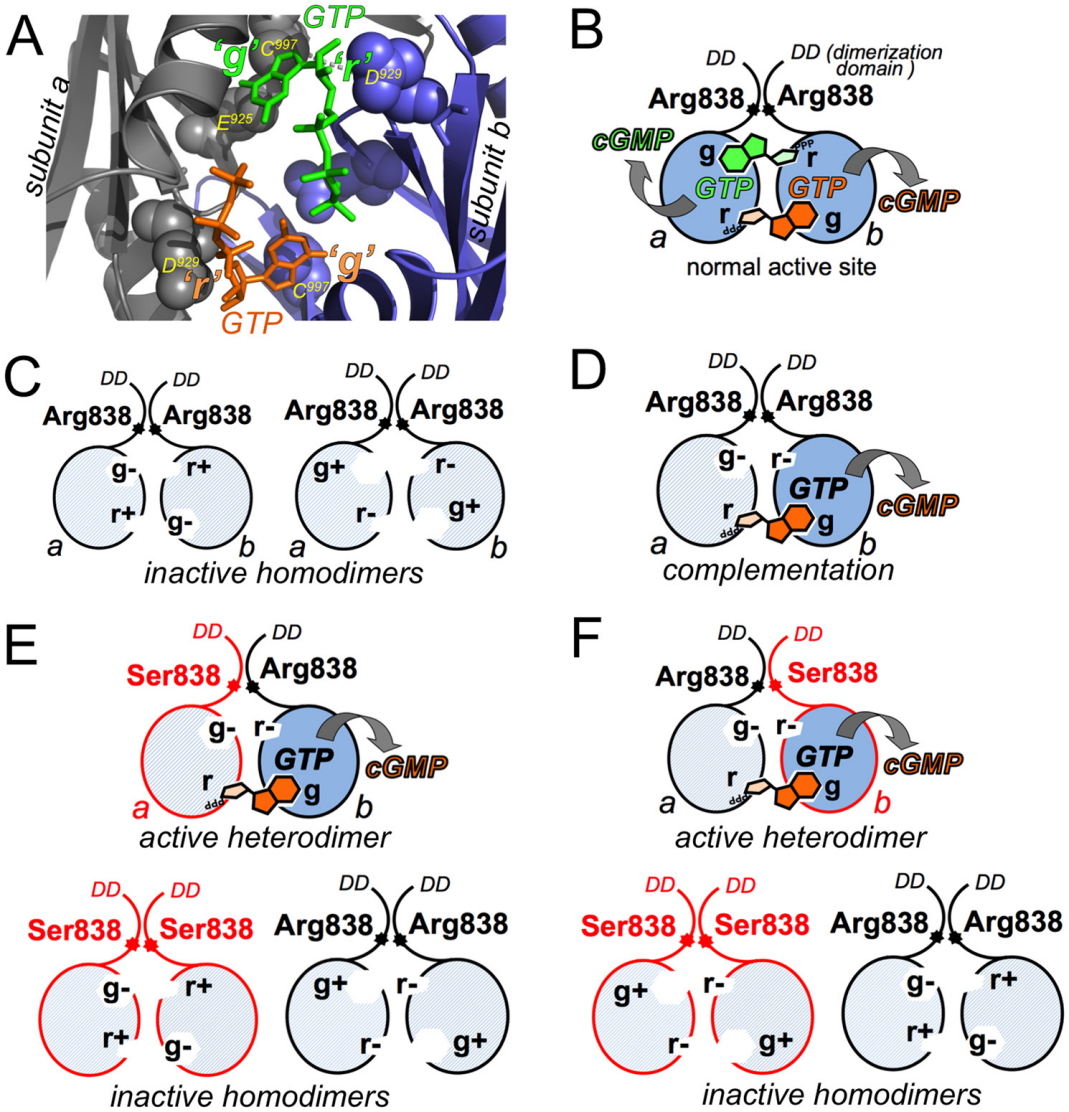
**A paradigm for measuring guanylyl cyclase activity in RetGC1 heterodimer Arg^838^:Ser^838^.***A*, three-dimensional structure of the active site in RetGC1 (52). The catalytic domains from two subunits, a (*gray*) and b (*blue*), coordinate two GTP molecules (*green* and *orange*), each guanine base, via Glu^925^ and Cys^997^ (the g site) and the Mg^2+^ ribose-5´-triphosphate moiety, via Asp^929^ (the r site). *B*, binding of each GTP molecule requires the g and r sites located on the opposite subunits. *C*, inactivation of a single g or r site (*g*^—^ or r^—^) prohibits the resultant homodimers from coordinating both GTP molecules (35, 45, 51). *D*, complementation between the (g^—^r^+^) and (g^+^ r^—^) subunits allows their heterodimer (g^—^r^+^):(g^+^ r^—^) to coordinate one GTP molecule in the active site (the subunit coordinating guanine base is shown with *dark shading*) (35, 45, 51). *E* and *F*, to selectively measure the activity of heterodimer Arg^838^:Ser^838^, the (g^+^ r^—^) subunit harboring WT Arg^838^ (*E*) or CORD6 Ser^838^ (*F*) in its dimerization domain is co-expressed with the complementing (g^—^r^+^) subunit that harbors, respectively, Ser^838^ (*E*) or Arg^838^ (*F*). Only the Arg^838^:Ser^838^ heterodimers in both cases can bind and convert GTP to cGMP, whereas the homodimers Arg^838^:Arg^838^ and Ser^838^:Ser^838^ are inactive.

R838S substitution has a dominant degenerative phenotype in CORD6 patients and in transgenic animals (33, 35, 37, 38). When the WT and R838S RetGC1 are co-expressed in a cell culture or in the retina, deceleration of the GCAP-stimulated RetGC activity requires higher than normal free Ca^2+^ concentrations (35, 36, 37, 38). However, it remains unclear whether the overall Ca^2+^ sensitivity decreases only because of the presence of the R838S RetGC1 homodimer or whether the heterodimers containing WT (Arg^838^) and CORD6 (Ser^838^) subunits also contribute to the change. Their co-expression produces the Arg^838^:Ser^838^ heterodimers mixed with Arg^838^:Arg^838^ and Ser^838^:Ser^838^ homodimers. Those dimers cannot be biochemically separated to test their individual Ca^2+^ sensitivities, largely because the detergents required for extraction of RetGC1 from the membrane destroy cyclase regulation (42). Instead, we tested the Ca^2+^ sensitivities in the Arg^838^:Ser^838^ heterodimers using a modification of the functional complementation method originally developed in J. Hurley's laboratory (35, 51) (Fig. 9).

In the RetGC1 catalytic domain structure (52), the active site of the cyclase binds two Mg^2+^ GTP molecules, each held by the opposite subunits via two different binding pockets (Fig. 9*A*). Each subunit (subunits a and b) coordinates the guanine base (the g site) of one GTP molecule and the ribose-5-triphosphate (the r site) of the other GTP molecule (Fig. 9, *A* and *B*). Substitutions E925K and C997D disable coordination of the guanine moiety (g*^—^*), and D929A disables coordination of the ribose-5-triposphate moiety (r*^—^*) (35, 51). Consequently, neither (g^—^ r^+^) nor (g^+^ r^—^) RetGC1 homodimer can bind GTP and produce cGMP (35, 51) (Fig. 9*C*). However, cGMP can be produced when (g^—^ r^+^) and (g^+^ r^—^) subunits are combined as a heterodimer. In this case, the remaining functional r site of the subunit a pairs with the remaining functional g site on the opposite subunit b (35, 45, 51) (Fig. 9*D*). We reasoned that the functional complementation between the Arg^838^ and Ser^838^ subunits (Fig. 9, *E* and *F*) should allow for selectively testing the Ca^2+^ sensitivity of GCAP1-activated heterodimers in the presence of the inactive homodimers.

**Figure 10:**
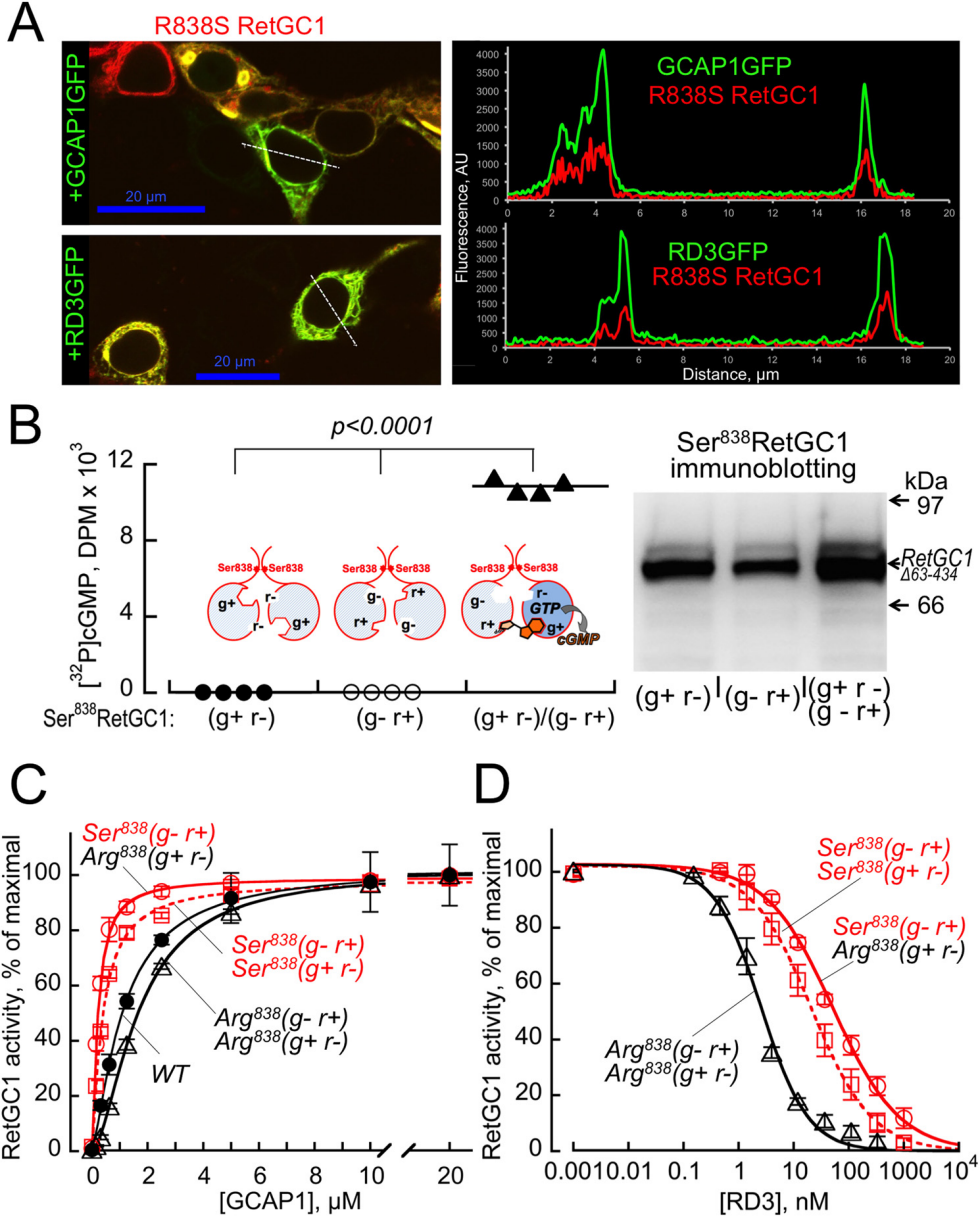
*A*, R838S substitution in RetGC1 does not prevent binding of GCAP or RD3 *in cyto*. R838S mOrange-RetGC1 was co-expressed with GCAP1-GFP (*top panel*) or RD3-GFP (*bottom panel*) in HEK293 cells as described under “Experimental procedures.” *Bars*, 20 μm. The distribution of two fluorochromes is shown in the *right panel*. PCC values are presented in Table 1, Table 2. *B*, neither Ser^838^ nor shorter extracellular domain prevent complementation between RetGC1 subunits. *Left panel*, Δ_63–434_ R838S RetGC1 variants (g^+^ r^—^) (*closed circles*) or (g^—^ r^+^) (*open circles*) are inactive when expressed separately but create the active dimer when co-expressed (*closed triangles*). *Right panel*, immunoblotting of the RetGC1 preparations used in the assay. *C*, RetGC1 activation by GCAP1 in 10 mm Mg^2+^ and 2 mm EGTA (means ± S.D., *n* = 3): WT (*closed black circles*), Arg^838^(g^—^ r^+^):Arg^838^(g^+^ r^—^) (*open black triangles*), Ser^838^(g^—^ r^+^):Ser^838^(g^+^ r^—^) (*open red squares*), and Ser^838^(g^—^ r^+^):Arg^838^(g^+^ r^—^) (*open red circles*) RetGC1. The data were fitted assuming a sigmoidal function, *A*_%_ = 100/(1 + ([GCAP]_1/2_/[GCAP])*^h^*), where *A*_%_ is a percentage of the maximal activity in each mutant, [GCAP] is the concentration of GCAP1 in the assay, [GCAP]_1/2_ is the half-saturating concentration of GCAP1 (1.1 ± 0.05, 1.65 ± 0.041, 0.40 ± 0.02, and 0.22 ± 0.02 μm, respectively), and *h* is the Hill coefficient. *D*, dose dependence of RetGC1 inhibition by RD3. The Arg^838^(g^—^ r^+^):Arg^838^(g^+^ r^—^) (*open black triangles*), Ser^838^(g^—^ r^+^):Ser^838^(g^+^ r^—^) (*open red squares*), and Ser^838^(g^—^ r^+^):Arg^838^(g^+^ r^—^) (*open red circles*) RetGC1 dimers in the assay were preactivated by 1.5 μm GCAP1 in the presence of 10 mm Mg^2+^ and 2 mm EGTA. The data (means ± S.D., *n* = 3) were fitted assuming a sigmoidal function, *A*_%_ = 100/(1 + ([RD3]/[RD3]_1/2_)*^h^*), where *A*_%_ is a percentage of the maximal activity in each mutant, [RD3] is the concentration of RD3 in the assay, *h* is the Hill coefficient, and [RD3]_1/2_ is the RD3 concentration causing 50% inhibition (2.3 ± 0.24, 23 ± 7, and 42 ± 1.5 nm, respectively).

We verified that the Ser^838^ in dimerization domains permitted the functional complementation between GCAP1-activated (g^—^ r^+^) and (g^+^ r^—^) RetGC1 subunits. The Ser^838^ did not prevent binding of GCAP1 (Fig. 10*A* and Table 1), but neither Ser^838^(g^—^ r^+^) nor Ser^838^(g^+^ r^—^) RetGC1 produced cGMP in the presence of Mg^2+^ GCAP1 when expressed separately. Only their co-expression enabled cGMP synthesis through the formation of the heterodimer (Fig. 10*B*), making it possible to compare the regulatory properties of different dimeric RetGC1 combinations (Fig. 9). Deletion of Ala^63^ through Met^434^ from the extracellular domain did not prevent the complementation (Fig. 10*B*). It is worth mentioning that the E925K/C997D substitutions enable binding of ATP instead of GTP via the g^—^ site (35, 45, 51). We only assessed the Ca^2+^ sensitivity of GTP to cGMP conversion, because high concentrations of Mg^2+^ATP as a substrate for adenylyl cyclase activity skew the Ca^2+^/EGTA buffering accuracy in the assay.

R838S substitution increases the affinity of RetGC1 for Mg^2+^GCAP1 (34, 35, 36, 37). It was also elevated in the Ser^838^(g^—^ r^+^):Ser^838^(g^+^ r^—^) heterodimer harboring a single GTP-binding site as compared with the Arg^838^:Arg^838^ dimers binding one or two GTP molecules (Fig. 10*C*). The apparent affinity of RetGC1 for Mg^2+^ GCAP1 was increased even further in the Ser^838^(g^—^ r^+^):Arg^838^(g^+^ r^—^) heterodimer (Fig. 10*C*), which recognized GTP via the Arg^838^ subunit (Fig. 9*E*). R838S substitution also did not prevent RD3 binding to RetGC1 *in cyto* (Fig. 10*A* and Table 2), but the deceleration of GCAP1-stimulated RetGC1 activity required higher concentrations of RD3 to inhibit the Ser^838^(g^—^ r^+^):Arg^838^(g^+^ r^—^) heterodimer than the Arg^838^:Arg^838^ and even the Ser^838^(g^—^ r^+^):Ser^838^(g^+^ r^—^) dimers (Fig. 10*D*).

**Figure 11:**
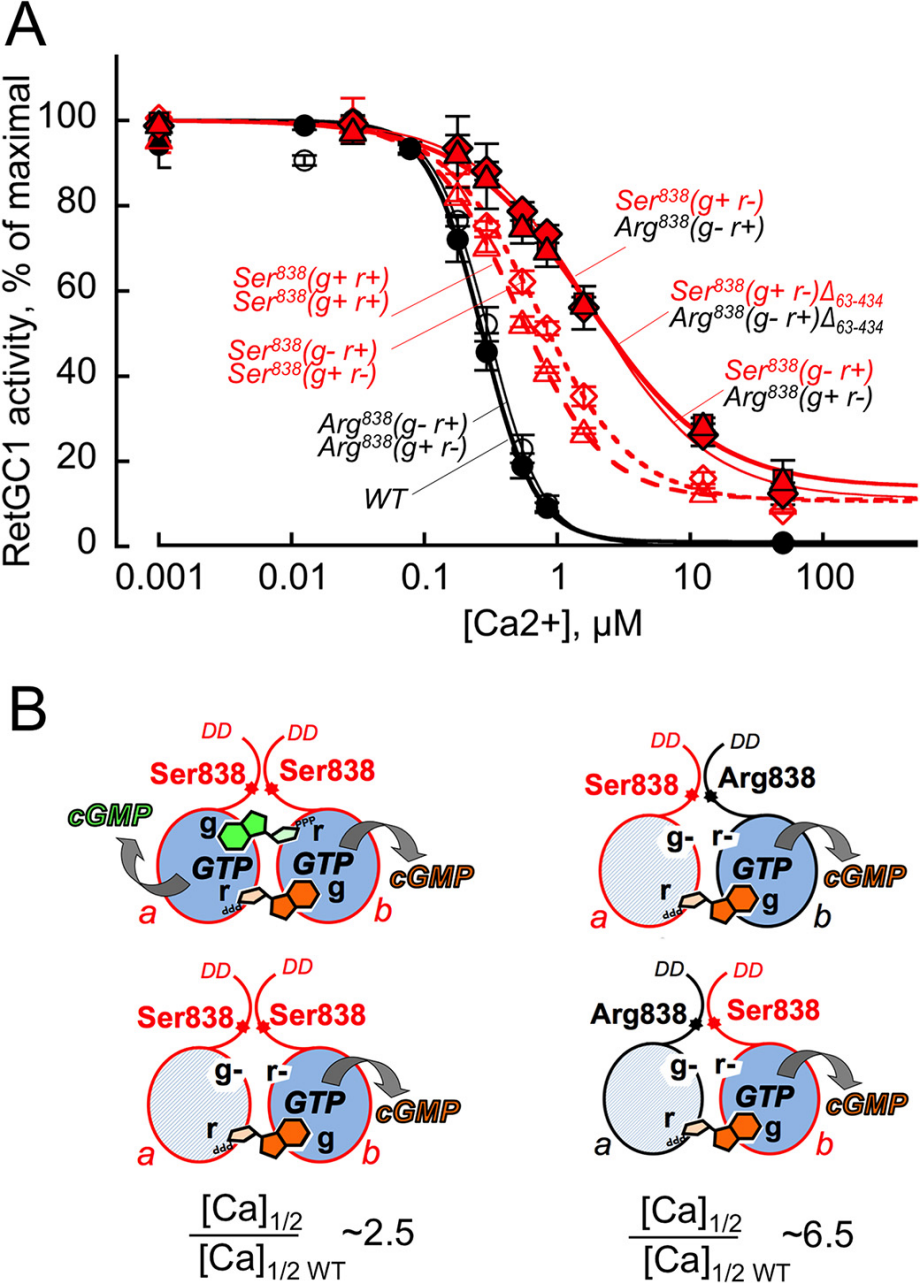
**The R838S substitution affects Ca^2+^ sensitivity in RetGC1 heterodimer Arg^838^:Ser^838^ even stronger than in homodimer Ser^838^:Ser^838^.***A*, Ca^2+^-dependent deceleration of the RetGC1 activity. WT RetGC1 (*filled circles*) and the dimeric combinations, Arg^838^(g^—^ r^+^):Arg^838^(g^+^ r^—^) (*open circles*), Ser^838^(g^+^ r^+^):Ser^838^(g^+^ r^+^) (*red open triangles*), Ser^838^(g^—^ r^+^):Ser^838^(g^+^ r^—^) (*red open diamonds*), Ser^838^(g^—^ r^+^):Arg^838^(g^+^ r^—^) (*red filled diamonds*), and Ser^838^(g^+^ r^—^):Arg^838^(g^—^ r^+^) (*red filled triangles*) or the Δ_63–434_ Ser^838^(g^+^ r^—^):Arg^838^(g^—^ r^+^) RetGC1 (*red filled squares*) were activated by 14 μm GCAP1 at 0.9 mm free Mg^2+^ in the presence of variable free Ca^2+^ concentrations; the data were fitted assuming a sigmoidal function, *A*_%_ = (100 − *A*_min_)/(1 + ([Ca]/[Ca]_1/2_)*^h^*) + *A*_min_, where *A*_%_ is a % of the maximal cyclase activity in each case, *A*_min_ is the activity at saturating Ca^2+^, [Ca] is the concentrations of free Ca^2+^ in the assay, and *h* is the Hill coefficient. The respective [Ca]_1/2_ values (means ± S.D., *n* = 3) were 0.27 ± 0.033, 0.305 ± 0.040, 0.507 ± 0.04, 0.717 ± 0.080, 1.84 ± 0.43, 1.70 ± 0.4, and 1.70 ± 0.39 μm. *B*, *left panel*, in the Ser^838^:Ser^838^ dimers, [Ca]_1/2_ is ∼2.5-fold higher than in WT (Student's *t* test, *p* < 0.0013). *Right panel*, [Ca]_1/2_ in Arg^838^:Ser^838^ heterodimers rises to ∼6.5-fold higher than in WT (*p* < 0.0001), regardless of which subunit in the dimer, the Arg^838^ or Ser^838^, binds the purine base of GTP.

In the Arg^838^(g^—^ r^+^):Arg^838^(g^+^ r^—^) RetGC1 that binds only one GTP molecule per active site (Fig. 9*C*), the Ca^2+^ sensitivity of its regulation by GCAP remained identical to the WT (Fig. 11*A*). In contrast, it was strongly reduced in the dimers harboring Ser^838^. The [Ca^2+^]_1/2_ in Ser^838^(g^+^ r^+^):Ser^838^(g^+^ r^+^) and Ser^838^(g^—^ r^+^):Ser^838^(g^+^ r^—^) dimers was increased ∼2.5-fold compared with the WT, also regardless of whether they bound two or just one GTP per active site (Fig. 11, *A* and *B*). The [Ca^2+^]_1/2_ increased even further, to ∼6.5-fold higher than the WT, in the Arg^838^:Ser^838^ RetGC1 heterodimers (Fig. 11, *A* and *B*). In contrast to the single-residue substitution, R838S, a deletion of ∼40-kDa fragment from the cyclase extracellular domain did not alter the Ca^2+^ sensitivity of the Ser^838^(g^+^ r^—^):Arg^838^(g^—^ r^+^) heterodimer (Fig. 11*A*), because the extracellular domain does not contribute to the regulation of RetGC1 by GCAP (57).

## Discussion

### GUCY2D CSNB alleles paradoxically code for inactive RetGC1

*GUCY2D* LCA1-linked substitutions in RetGC1 disable rod and, even more severely, cone function, yet the vast majority of the photoreceptors remain alive (39). The residual physiological rod, but not cone, responses can be detected in RetGC1-deficient retinas (5, 39), most likely because of the presence of a rod-specific (6) RetGC2(*GUCY2F*) isozyme (2, 3, 7, 48), which does not form a heterodimer with RetGC1 *in vivo* (53). RetGC2 regulated by GCAP2 provides a smaller-scale ancillary cGMP production (7, 45), helping to accelerate rod recovery from excitation (47, 48, 54). In contrast, cones would normally produce cGMP using almost exclusively GCAP1-regulated (and possibly GCAP3-regulated) RetGC1 (6, 14, 50, 55). Therefore, the loss of RetGC1 activity documented previously (39, 44, 45, 46) and in the present study (Fig. 6) can explain the main biochemical reasons for the loss of function in *GUCY2D* LCA1 photoreceptors reasonably well.

The role of *GUCY2D* mutations in selectively suppressing rod vision (40) is much more difficult to explain. Instead of finding biochemical differences in RetGC1 regulation by GCAPs and RD3, potentially explaining the unusual physiology of the affected retinas, we documented that CSNB *GUCY2D* alleles (40) merely code for biochemically inactive RetGC1 (Figure 6, Figure 7, Figure 8). The low residual activity detectable in GCAP1-stimulated R666W RetGC1 (Fig. 6*A*) was similar to that of previously characterized LCA1 mutant R1091X (39), and its apparent affinity for GCAP1 was even 7-fold lower than in R1091X (39). The two other tested CSNB mutants were completely inactive (Fig. 6). The L911F RetGC1 retains the ability to recognize and bind GCAP1 *in cyto* (Figs. 3*C* and 8 and Table 1), but even in the presence of GCAP1, it lacks catalytic activity (Fig. 6*A*). Activation by GCAP3, expressed exclusively in a subset of human cones (50), would have helped to explain the selective preservation of cone vision in CSNB, but none of the tested CSNB mutants was stimulated by GCAP3 (Fig. 6*C*).

Hypothetically, RetGC1 in CSNB photoreceptors could acquire activity through complementation with the accompanying products of LCA1 alleles (40). However, co-expression of CSNB RetGC1 with their respective LCA1 mutants failed to produce active enzyme (Fig. 8). Evidently, CSNB and LCA1 RetGC1 are unable to form heterodimers, or such heterodimers remain nonfunctional. The loss of the residual activity in R666W (Fig. 6*A*) after its co-expression with G982VfsX39 (Fig. 8) may reflect the formation of a nonfunctional heterodimer in which the truncated catalytic domain of the LCA1-specific subunit completely disabled the active site.

We found yet another deficiency in two CSNB/LCA1 allelic combinations, L911F/R768W and R761W/R768W (40), which would also likely make them nonfunctional *in vivo*; none of the individual mutants in these combinations effectively binds RD3 (Figure 3, Figure 4, Figure 5 and Table 1, Table 2). The delivery of the cyclase in the outer segment requires RD3 (21, 24, 25); therefore, the mutated RetGC1 would be unlikely to effectively reach the outer segment.

Based on the biochemical properties observed in this study, one would expect that the *GUCYD* CSNB mutations would be more likely to cause LCA1-like rod and cone blindness than to selectively preserve cone vision (40). Two hypothetical scenarios could conceivably reconcile the biochemical and clinical phenotypes: (i) some presently unknown modification(s) rescue the activities of CSNB RetGC1 variants in cones but not in rods or (ii) RetGC2 in those CSNB patients is strongly expressed in cones and produces cGMP instead of RetGC1. Neither possibility, however, can be experimentally tested, which currently leaves the field with a paradox, because the biochemistry of RetGC1 regulation directly contradicts the physiology of the *GUCY2D* CSNB photoreceptors.

### Ca^2+^ sensitivity of R838S RetGC1 defines the CORD6 dominant phenotype

The gain-of-function substitutions of Arg^838^ cause a dominant degenerative phenotype by hampering RetGC1 deceleration at the Ca^2+^ concentrations typical for dark-adapted photoreceptors (34, 35, 36, 37). The Ca^2+^ sensitivity of the RetGC1–GCAP complex is reduced because the affinity of RetGC1 for Mg^2+^GCAP increases as a result of the mutation (34, 35, 36). We find that the guanylyl cyclase activity in heterodimers comprised of the Arg^838^- and Ser^838^-containing subunits becomes even less sensitive to the inhibition by Ca^2+^ than in the Ser^838^:Ser^838^ homodimer (Fig. 11). The very high Arg^838^:Ser^838^ heterodimer resistance to deceleration by Ca^2+^, even stronger than in the Ser^838^:Ser^838^ homodimer (Fig. 11*A*), is consistent with the additional increase of the heterodimer's affinity for Mg^2+^ GCAP1 (Fig. 10*C*).

Mutational analyses of RetGC1 indicate that its dimerization domain and the kinase homology domain are most critical for GCAP binding (44, 45). Previous studies of R838S RetGC1 (35, 36, 37) suggested that its Ser^838^:Ser^838^ coiled-coil dimerization domain is more optimal than WT for the regulatory binding of GCAP1. We find in our study that pairing WT and CORD6-specific dimerization domains is even more conducive to GCAP1 binding.

To summarize, the heterodimers formed in photoreceptors that harbor heterozygous CORD6 *GUCY2D* allele would likely stimulate Ca^2+^ influx in the dark even more than Ser^838^:Ser^838^ homodimers. It would therefore seem unreasonable to expect that decreasing the fraction of the Ser^838^:Ser^838^ homodimers by increasing the level of WT *GUCY2D* allele expression instead of reducing the expression of the CORD6 allele could alleviate photoreceptor degeneration.

### Possible role of RD3 in GUCY2D CORD6 pathology

RD3 suppresses RetGC–GCAP complex activity by displacing GCAP from the cyclase (22, 23, 37). RetGC1 binds RD3 via interface that is nonidentical to the GCAP-binding site(s) (58, 59, 60). The C-terminal portion of RetGC1 is critical for binding RD3 (24) but not GCAPs (24, 45). Consistent with that, the G982VfsX39 and L911F RetGC1 can bind GCAP1 but not RD3 *in cyto* (Figs. 2*C* and 3*C*, Table 1, Table 2). Conversely, the cyclase dimerization domain is more critical for GCAP binding than for RD3 binding (44, 45). On the other hand, some of the disease-linked substitutions in the cyclase kinase homology domain (R761W and R768W) can compromise both GCAP1 and RD3 binding (Figure 4, Figure 5), thus suggesting that the GCAP-binding and RD3-binding interfaces on the cyclase could be partially overlapped or affect each other in the quaternary structure of the complex. The increased affinity for Mg^2+^ GCAP1 evidently causes the Arg^838^:Ser^838^ heterodimer to require higher RD3 concentrations for its inhibition than the WT RetGC1 homodimer and even higher than the Ser^838^:Ser^838^ homodimer (Fig. 10*C*). *In vivo* studies strongly indicate that one of the essential RD3 functions in photoreceptors prevents photoreceptor degeneration by suppressing aberrant guanylyl cyclase activation by GCAPs (27), most likely in the inner segment (27, 61). Consequently, the increased resistance of the heterodimers to inhibition by RD3 would likely contribute to the severity of CORD6 retinal degeneration.

## Experimental procedures

### Materials

Unless specified otherwise, nucleotides were purchased from Millipore/Sigma, chemicals (ultrapure or molecular biology grade) were from Millipore/Sigma or Fisher Scientific, restriction endonucleases were from New England Biolabs, Phusion Flash polymerase was from Thermo Scientific, and oligonucleotide primers were from Integrated DNA Technologies.

### RetGC1 mutagenesis, expression, and activity assays

Human RetGC1 (G*UCY2D*) cDNA inserted in pRCCMV vector (Invitrogen) was modified to introduce new restriction sites for subsequent cloning without changing the encoded protein sequence. LCA1-, CSNB-, and CORD6-specific substitutions were then introduced using “splicing by overlap extension” method (62). DNA fragments amplified in a Phusion Flash DNA polymerase mixture were purified using a ZymoResearch DNA Cleanup kit, digested at the ends with the appropriate restriction endonucleases, and ligated in the RetGC1 cDNA part of the plasmid. The resultant plasmids were isolated using a Promega Wizard protocol and verified by sequencing on both strands. Where indicated, the mOrange tag (Clontech) cDNA was inserted, substituting in-frame a ∼26-kDa portion of the RetGC1 “extracellular” domain, downstream from the leader peptide and upstream from the transmemebrane domain (44, 45). RetGC1 variants were expressed in HEK293 cells transfected using calcium-phosphate precipitation method (44, 58), and the membrane fractions containing the recombinant RetGC1 were isolated as previously described (44). The guanylyl cyclase activity was assayed as previously described in detail (36) with the modifications described in Ref. 59. In brief, the assay mixture (25 μl) containing HEK293 membranes, 30 mm MOPS–KOH, pH 7.2, 60 mm KCl, 4 mm NaCl, 1 mm DTT, 2 mm EGTA, or 2 mm Ca^2+^/EGTA buffers, Mg^2+^ as indicated in experiments, 0.3 mm ATP, 4 mm cGMP, 1 mm GTP, and 1 μCi of [α–^32^P]GTP (PerkinElmer), 100 μm zaprinast and dipyridamole was incubated for 30 min at 30 °C, and the reaction was stopped by heat-inactivation for 2 min at 95°. The [^32^P]cGMP product was separated by TLC using fluorescently backed polyethyleneimine cellulose plates (Merck) developed in 0.2 m LiCl. The cGMP spots visualized under UV light were cut out from the plate, and the [^32^P]cGMP was eluted with 0.5 ml of 2 m LiCl in 20-ml scintillation vials. The radioactivity was counted by liquid scintillation in 10 ml of UniverSol (MP Biochemicals). Data fitting was performed using Synergy Kaleidagraph 4 software.

### Immunoblotting

HEK293 cell fractions containing the recombinant RetGC1 were subjected to electrophoresis in 7% SDS-PAGE and transferred to Immobilon-P membrane (Millipore Sigma) at 60 V for 20 h. The membranes were blocked using SuperBlock blend (Fisher Scientific), and probed by rabbit polyclonal antibody raised against the recombinant C-terminal fragment, Met^748^–Ser^1103^, of a human RetGC1 (RRID:AB_2877058), and the chemiluminescence image developed using a Pierce SuperSignal Femto kit was acquired using a Fotodyne Luminous FX instrument.

### RD3 expression and purification

The recombinant human RD3 was expressed from pET11d (Novagen/Calbiochem) vector in BL21(DE3) CodonPlus *Escherichia coli* strain (Agilent Technologies), extracted from the inclusion bodies, and purified as previously described (22, 59) with the modifications described in Ref. 60. The purity of the preparations was verified by SDS-PAAG electrophoresis, Coomassie Blue staining, and densitometry. The protein solution was mixed with glycerol to final 35% (v/v), then aliquoted, frozen in liquid N_2_, and stored in −70 °C. The aliquots were thawed only once, immediately before use in the RetGC assay.

### GCAP expression and purification

Human GCAP1 (E6S), GCAP2, and GCAP3 cDNAs were inserted into the NcoI/BamHI sites of the pET11d vector (originated from Novagen/Calbiochem). The *N*-myristoylated GCAPs for the *in vitro* assays were expressed in BLR(DE3) *E. coli* strain harboring pBB131 plasmid coding for a yeast *N*-myristoyl transferase and urea-extracted from the inclusion bodies. GCAP1 was purified by calcium precipitation, butyl–Sepharose chromatography, and Sephacryl S–100 chromatography using previously published procedure (58) modified as described in Ref. 64. The purity of GCAPs preparations verified by SDS gel electrophoresis was ≥90%. The human GCAP2 was purified as previously described (65) with the following modifications. The cells were grown in 0.5 liter of standard LB medium containing 50 μg/ml kanamycin and 100 μg/ml ampicillin until they reached *A*_600_ 0.6–0.7. Myristic acid was added from a concentrated ethanol solution to the suspension of bacterial cells to the final concentration of 100 μg/ml, 30 min prior to the induction with 0.5 mm isopropyl-β-d-thiogalactopyranoside. 3 h after the induction, the bacterial pellet was harvested by centrifugation at 8,000 × *g* for 10 min 4 °C, and frozen in −70 °C. The pellet was thawed; resuspended in 25 ml of 10 mm Tris-HCl, pH 7.5, containing 2 mm EDTA and 14 mm 2-mercaptoethanol; and sonicated on ice for 2 min. The inclusion bodies from the disrupted cells were collected by centrifugation at 20,000 × *g* for 20 min, 4 °C. The sonication/centrifugation step was repeated two more times. GCAP2 was extracted from the pellet by homogenization in 10 ml of 10 mm Tris-HCl, pH 7.5, containing 2 mm EGTA, 14 mm 2-mercaptoethanol, 2 mm MgCl_2_, and 8 m urea for 30 min at 4 °C. After centrifugation at 20,000 × *g* for 20 min at 4 °C, the supernatant was dialyzed at 4 °C, first for 3–4 h against 2.0 liters of 10 mm Tris-HCl buffer, pH 7.5, containing 0.5 mm EGTA, 1 mm MgCl_2_, and 7 mm 2-mercaptoethanol, and then overnight against 2 liters of 10 mm Tris-HCl, pH 7.5 buffer containing 0.1 mm EGTA, 1 mm MgCl_2_, and 7 mm 2-mercaptoethanol. After the dialysis, the insoluble material was removed by centrifugation at 20,000 × *g* for 20 min at 4 °C, and Tris-HCl and CaCl_2_ were added to the supernatant fraction to 50 mm and 10 mm, respectively. After 20 min at room temperature, the precipitated material was removed by centrifugation at 20,000 × *g* for 20 min at 4 °C, and the supernatant was collected, concentrated to 5 ml using Amicon Ultra-15 (10,000 molecular weight cutoff) centrifugal filter and then centrifuged for 10 min at 200,000 × *g* at 4 °C in a Beckman Optima ultracentrifuge and loaded on a Sephacryl S-100 column (2.6 × 60 cm) equilibrated with 20 mm Tris-HCl, pH 7.5, containing 100 mm NaCl and 0.3 mm CaCl_2_. After the main peak containing GCAP2 was collected, dithiothreitol was added to 4 mm and EDTA was added to 2 mm to remove Ca^2+^ bound to GCAP2. The excess of EDTA was then removed by 3–4 cycles of 20-fold concentration/dilution in 10 mm Tris-HCl, pH 7.5, containing 30 μm EDTA using an Amicon Ultra-15 (10,000 molecular weight cutoff) centrifugal filter at 4 °C. Human GCAP3 was purified using similar procedure except that 1.0 × 30-cm Superdex-200 column equilibrated with 20 mm Tris-HCl, pH 7.5, and 100 mm NaCl was used for size-exclusion chromatography instead of Sephacryl S-100.

### Ca^2+^/EGTA buffers

The EGTA/CaCl_2_/MgCl_2_ mixtures maintaining required free Ca^2+^ concentrations at 1 mm free Mg^2+^ were prepared using Tsien and Pozzan method (66) and verified by fluorescent indicator dyes as previously described in detail (63).

### Co-transfection and confocal imaging

The DNA transfection followed a Promega FuGENE protocol by the manufacturer as described in Refs. 45, 59, and 60. The HEK293 cells were transfected after reaching 30–50% confluence in a LabTeck 4-well cover glass chamber with the mixtures of bGCAP-GFP or hRD3-GFP coding plasmids with mOrange-RetGC1 coding plasmid, at ∼1/100 molar ratio (58, 59, 60), using 3 μl of Promega FuGENE reagent per 1 μg of DNA. Confocal images were taken after 24–30 h of incubation in 5% CO_2_ at 37 °C, using an Olympus FV1000 Spectral instrument with the respective 543- and 488-nm excitation for the red and the green fluorochromes in sequential mode. The images were processed, and the PCC values in whole-cell images were determined using Olympus FluoView FV10-ASW software as previously described (43, 44, 45, 58, 59, 60). No changes to the original images were made except for minor γ correction applied to whole image for more clear presentation in print. Quantitative analyses were performed using the original images without corrections.

### Statistics

Statistical significance of the differences was tested by ANOVA/Bonferroni post hoc at 99% confidence level or by unpaired/unequal variance Student's *t* test using a Synergy Kaleidagraph 4 software.

### Three-dimensional molecular visualization

The image of the RetGC1 active site was created using PyMOL molecular graphics system (version 2.0, Schrödinger, LLC) utilizing coordinates of the model structure reported by Liu *et al*. (52) (DOI 10.5452/ma-cps37).

## Data availability

The data referred to in this article are contained within the article. Unprocessed data can be available from the corresponding author (adizhoor@salus.edu) upon reasonable request.

10.13039/100000053HHS | NIH | National Eye Institute (NEI) (EY011522) to Alexander M. Dizhoor
10.13039/100004897Pennsylvania Department of Health (CURE Formula Grant) to Alexander M. Dizhoor
